# Domestic Environment and Gut Microbiota: Lessons from Pet Dogs

**DOI:** 10.3390/microorganisms10050949

**Published:** 2022-04-30

**Authors:** Juan Hernandez, Soufien Rhimi, Aicha Kriaa, Vincent Mariaule, Houda Boudaya, Amandine Drut, Amin Jablaoui, Héla Mkaouar, Amel Saidi, Vincent Biourge, Mohamed Ali Borgi, Moez Rhimi, Emmanuelle Maguin

**Affiliations:** 1 Microbiota Interaction with Human and Animal Team (MIHA), Micalis Institute, AgroParisTech, Université Paris-Saclay, Institut National de Recherche Pour l’Agriculture, l’Alimentation et l’Environnement, 78 350 Jouy-en-Josas, France; juan.hernandez@oniris-nantes.fr (J.H.); soufienrhimi@yahoo.fr (S.R.); aicha.kriaa@inrae.fr (A.K.); vincent.mariaule@inrae.fr (V.M.); boudayahouda12@gmail.com (H.B.); amandine.drut@oniris-nantes.fr (A.D.); amin.jablaoui@inrae.fr (A.J.); hela.mkaouar@yahoo.com (H.M.); saidiamel04@gmail.com (A.S.); emmanuelle.maguin@inrae.fr (E.M.); 2 Oniris, Department of Clinical Sciences, Nantes-Atlantic College of Veterinary Medicine and Food Sciences, 44 300 Nantes, France; 3 Royal Canin Research & Development Center, 30 470 Aimargues, France; vincent.biourge@royalcanin.com; 4 Laboratory of Biotechnology and Biomonitoring of the Environment and Oasis Systems, University of Gafsa, Gafsa 2112, Tunisia; borgima@yahoo.com

**Keywords:** pets, dogs, domestic environment, gut microbiota, animal model, microbiome, holobiont

## Abstract

Accumulating data show the involvement of intestinal microbiota in the development and maintenance of numerous diseases. Many environmental factors influence the composition and function of the gut microbiota. An animal model subjected to the same environmental constraints that will allow better characterization of the microbiota–host dialogue is awaited. The domestic dog has physiological, dietary and pathological characteristics similar to those of humans and shares the domestic environment and lifestyle of its owner. This review exposes how the domestication of dogs has brought them closer to humans based on their intrinsic and extrinsic similarities which were discerned through examining and comparing the current knowledge and data on the intestinal microbiota of humans and canines in the context of several spontaneous pathologies, including inflammatory bowel disease, obesity and diabetes mellitus.

## 1. Introduction

It is estimated that there are 100 trillion microorganisms in the human body containing more than 11 million genes [1]. Most of these microbial cells reside within the gut and have a profound influence on physiology [2]. Causal links between several noncommunicable chronic diseases and gut microbiota have now been established using animal models [3,4]. Mice have been chosen as a model to study nutritional impacts, the development of illnesses, and the effects of antimicrobials. Aiming to extrapolate such an understanding from mice to humans, the commonalities and disparities between their gut microbiota were reviewed [5,6]. Many differences between the physiologies of the mouse and human intestinal and immune systems have now been identified [7]. Comparative genomic studies show that the immune system and its regulatory pathways underwent major changes throughout evolution, demonstrating the host-specific adaptation of the gastrointestinal immune system [8]. Furthermore, wild mice are vegetarian omnivores, while humans are historically omnivorous, and only 4% of the microbial genes of the mouse microbiota catalog share at least 95% sequence identity with the human microbiota catalog [6,9]. The two datasets exhibit partial functional overlap. It is therefore essential to expand such studies to other animal models before extrapolating the findings to humans.

**Figure 1:**
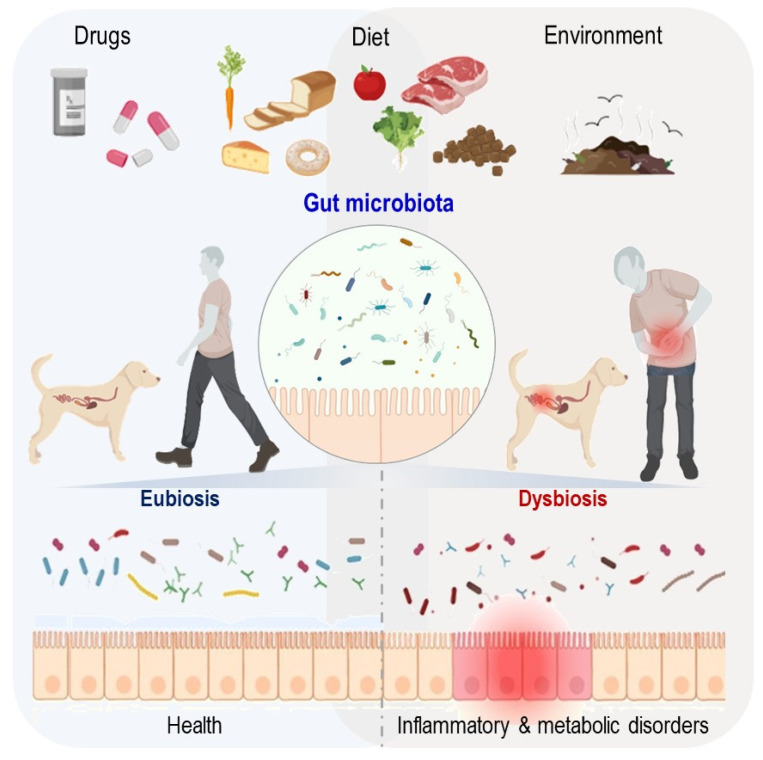
Overview of the impact of the domestic environment on the gut microbiota and health of humans and pet dogs.

The domestic dog, *Canis lupus familiaris*, is regarded as the first domesticated animal [10]. The dog provides a large animal model that is more comparable to humans than mice from physical and clinical perspectives (Table 1). Indeed, the domestic dog has an omnivorous metabolism and can digest, absorb and metabolize dietary carbohydrates [11]. More importantly, pet dogs also share their owners’ environments and are hence affected by their “lifestyles” in addition to their own genetic traits. Many naturally occurring canine diseases have similar human counterparts, notably noncommunicable chronic disorders such as chronic inflammatory diseases, diabetes mellitus and obesity [12] (Figure 1). Additionally, a comparison of the canine and human microbiota reveals both commonalities and discrepancies [13]. When comparing the human gut microbiota gene catalog to the catalogs of swine, mice and dogs built with datasets obtained via similar technology, the canine gastrointestinal microbiota has the highest taxonomic and functional overlap with the human intestinal microbiota [6,7,14,15]. In this review, we provide a concise analysis of the domestic environment shared by humans and pet dogs, and we focus on comparing the composition and function of the gut microbiota of both species in addition to the relevance of the canine model for studying interactions between the gut microbiota and its host.

## 2. Pet Dogs Share Domestic Environments with Their Owners

### 2.1. Domestic Dogs’ Lifestyles

The domestic dog and humans share a long common history that has intensified over time to the point of the dog becoming “man’s best friend”. Today, we can distinguish, on the one hand, the pet dog of industrialized countries, which lives in one’s home and occupies the full place of a family member, and on the other hand, the stray dog of developing countries, which can be stray or semi-stray. The former is in daily contact with its owner and shares resting and cooking areas, which promotes the transmission of commensals and pathogens in both directions [16]. Its food is mainly industrially composed of sources of protein, lipids and cooked starch. Its health is closely monitored, which exposes it to the same constraints of hygiene and medicalization (vaccination, antiparasitic drugs, etc.) as the humans around it. In 2018, 34% of American households owned a pet dog [17].

### 2.2. Domestic Environmental Exposure

The pet dog shares the domestic environment of its owners and is therefore exposed to the same environmental factors. Some studies tend to show that the owners of dogs affected by noncommunicable diseases are more prone to said diseases than those with disease-free dogs. Delicano et al. reported an increased risk of type 2 diabetes in the owners of diabetic dogs compared to those of nondiabetic dogs, supporting their role as sentinels of shared diabetogenic health behaviors or environmental exposures [18]. Previously, Glickman et al. reported a higher risk of mesothelioma in dogs whose owners are exposed to asbestos, confirming the sentinel role of the pet dog in identifying environmental health hazards for humans [19]. This sentinel role has also been demonstrated for exposure to various environmental toxins, such as diethylhexyl phthalate, polychlorinated biphenyl 153 and lead [20,21].

In addition, several diseases are common to pet dogs and humans, including metabolic disorders (diabetes mellitus, obesity, etc.), cardiovascular diseases (systemic arterial hypertension, etc.), chronic inflammatory diseases (inflammatory bowel disease, chronic bronchitis, etc.), neuropsychiatric diseases, and neoplastic diseases [13]. Recently, Yaglom et al. detected cross-species SARS-CoV-2 transmission between humans and dogs [22]. Veterinary therapies for dogs are similar to those for humans, so pet dogs are subjected to the same pharmacopeia used in humans [12]. Some of these diseases are favored by factors clearly identified in humans that are shared by their pets, such as eating, physical activity habits and exposure to xenobiotics.

### 2.3. Pet Dogs’ Diets

Like humans, pet dogs are considered omnivores [23]. After domestication, the dog made a transition from a carnivorous diet, facilitated by hunting, to an industrialized omnivorous diet higher in fiber and starch [11]. Recent studies have shown that three genes (AMY2B, MGAM and SGLT1) involved in the digestion of starch and in the uptake of glucose have been positively selected during dog domestication and are considered to represent evolutionary adaptation to their modern starch-rich diet. As is the case in humans, dogs digest starch very well, with an apparent ileal digestibility greater than 90% and a proportion of resistant starch available for the colonic microbiota [24,25,26]. Other recent studies showed that certain metabolic characteristics of the dog, such as the ability to synthesize enough niacin, taurine, and arginine, make it more similar to omnivores such as humans [27].

The pet dog provides valuable insights, as it shares the home environment, diet and eating habits, as well as spontaneous diseases and therapies of its owner. We can therefore anticipate that the environmental factors that interact with the human gut microbiota could have similar effects on the gut microbiota of pet dogs.

## 3. Pet and Human Gut Microbiota in Health Conditions

### 3.1. From Birth to Adulthood

It has been widely documented, both in pets and humans, that the composition of the gut microbiota changes over time.

In dogs, the bacterial composition of the fecal microbiota shows significant interindividual variability. At the age of 2 days, it was mainly represented by 29–95% Firmicutes, followed by Proteobacteria and Fusobacteria [28]. At the age of 2 months, the fecal microbiota presented a higher diversity at the phylum level, with a predominance of Bacteroidetes, followed by Firmicutes, Fusobacteria and Proteobacteria [28]. At this age, the fecal microbiota profile was markedly different from that of bitches, predominantly represented by Firmicutes, Fusobacteria and Bacteroidetes [28]. A significant depletion in the representation of the *Bifidobacterium* genus was identified in adult and senior dogs compared to puppies [2].

Early studies of human neonatal development showed that the gut microbiota starts to exhibit adult-like characteristics by the age of three years, but recent studies have suggested that its complete development may take longer [29,30]. Many factors not yet studied in pet dogs influence the composition of the human gut microbiota of a newborn and its evolution, such as the mode of feeding (breastfeeding or formula feeding) and delivery (natural delivery or C-section) [31]. The gut microbiota of 0–1- and 1–6-month-old groups are characterized by low biodiversity and are mostly represented by two main phyla, Actinobacteria (*Bifidobacterium* genus) and Proteobacteria. Firmicutes are poorly represented at this age, in contrast to pet dogs. However, the humans of 6–36 months are characterized by the presence of the *Bacteroides*, *Faecalibacterium*, *Blautia* and *Ruminococcus* genera, which are typical of adult microbiota [31]. The bacterial species found in adult human microbiota mostly belong to Proteobacteria, Firmicutes, Actinobacteria and Bacteroidetes.

These observations give rise to the hypothesis that the bifidobacterial community shows comparable trends in the canine and human gut microbiota [2,30].

### 3.2. Gut Microbiota along the Gastrointestinal Tract

It is widely known that the gut microbiota varies along the gastrointestinal tract [32]. The particularity of each region of the GI tract, such as the acidic nature of the stomach, the profile of bile acids and enzyme richness in the small intestine and the low oxygen availability in the colon, have a great impact on which microbiota species colonize each segment [32,33]. In dogs, most reports focus on the analysis of fecal microbiota due to the practical difficulties and ethical constraints related to sample collection from each intestinal compartment in privately owned animals [28,29]. Suchodolski et al. evaluated the microbial communities of the duodenal, jejunal, ileal and colonic digesta of healthy dogs through 16S rRNA gene analysis [33]. Firmicutes, Fusobacteria, Bacteroidetes and Proteobacteria were the predominant phyla from the four sites, and there was a gradual increase in bacterial diversity along the gastrointestinal tract from the duodenum to the colon [30]. Similarly, Honneffer et al. analyzed the contents of the duodenum, ileum, colon and rectum from six healthy dogs via Illumina sequencing of 16S rRNA genes [34]. In addition to the four previously mentioned phyla, Actinobacteria was identified but contributed minimally in each segment [32].

For humans, there are only a few studies describing the bacterial biogeography of the entire GI tract. Vuik et al. characterized the mucosal microbiota along the entire GI tract using mucosal biopsies taken from nine different regions from the distal esophagus to rectum in 14 individuals [35]. The upper GI tract was dominated by Proteobacteria and Firmicutes, followed by Bacteroidetes and Actinobacteria at low concentrations. However, in the lower GI tract, the representation of Proteobacteria consistently decreased, while that of Firmicutes increased, and they dominated the large intestine in the distal colon, followed by Bacteroidetes, which had become a dominant phylum [32,33,34,35].

**Figure 2:**
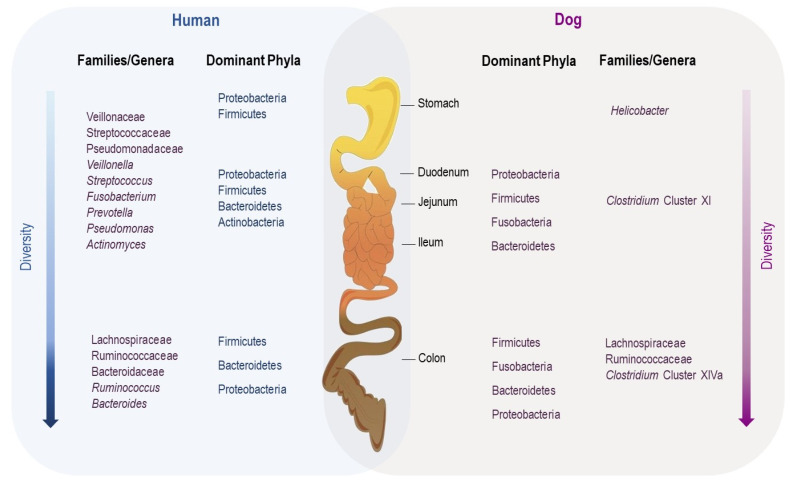
Comparative biogeography of the gut microbiota.

Although it is difficult to compare human and canine data due to methodological differences, the examination of the horizontal distribution of the gut microbiota reveals similarities and differences between the two species (Figure 2). Further studies using a standardized methodology are needed in the future.

### 3.3. Effects of Diet

In both humans and their pet dogs, diet is considered a key factor that influences the gut microbiota structure and host metabolic functions [36,37]. Evidence shows that the gut microbiota evolves to adapt to high intakes of fiber, carbohydrate and proteins. Several studies have compared the effects on the gut microbiota of feeding dogs with bones and raw food (BARF) diets (uncooked meat and bones) vs. commercial food (nutritionally balanced for omnivores, with a high abundance of fibers and carbohydrates). Bermingham et al. and Schmidt et al. demonstrated an overall decrease in the relative abundance of Firmicutes and Bacteroidetes involved in the digestion of dietary fibers in dogs fed with BARF diets [38,39]. They also identified a higher abundance of Proteobacteria, *Fusobacterium*, *Lactobacillus* and *Clostridium*. Most of the bacteria that decreased in abundance are associated with the production of short-chain fatty acids (SCFAs) from dietary carbohydrates, indicating a decrease in the fermentation of carbohydrates due to a decrease in carbohydrate intake [40]. Alessandri et al. showed a gradual rise in the relative abundance of bacterial taxa such as *Prevotella* and *Sutterella*, which break down carbohydrates, and a notable decline in the relative abundance of *Parabacteroides* and Ruminococcaceae in dogs fed with commercial food compared to dogs fed with a BARF diet [2].

These data mirror those for humans, especially when we compare Mediterranean-style with Western-style diets. While the Mediterranean diet (MD) is characterized by a high amount of dietary fiber, the Western diet (WD) is rich in animal protein and saturated fat [41,42]. In the majority of published studies, the gut microbiota composition differs between those consuming MD- and WD-style diets [41]. For example, high levels of *Bacteroides* sp. are found in WD-style diets, while *Prevotella* sp. have been observed to increase under an MD diet [41,43]. Shankar et al. demonstrated that the differences in gut metabolites and microbial composition and functions between Egyptian and US children are consistent with their diets [43]. While the gut microbiota of Egyptian children was found to be characterized by higher levels of SCFAs and increases in several polysaccharide-degrading microbes and end-products of polysaccharide fermentation, the gut microbiota of US children was found to have increased proteolytic microbes and end-products of protein and fat metabolism [43].

### 3.4. Effects of Xenobiotics

Pet dogs benefit from advanced veterinary care and are treated with the same classes of drugs as humans (antibiotics, anticancer chemotherapeutic drugs, proton-pump inhibitors, anti-inflammatory agents, vaccines, etc.). Therefore, pet dogs can contribute to studies of the impact of exposure to these substances on gut microbiota and health throughout life.

In recent years, several studies have evaluated the effects of certain xenobiotics on the gut microbiota of humans and pet dogs. The administration of omeprazole (a proton-pump inhibitor (PPI)) to healthy dogs results in higher proportions of Firmicutes and Fusobacteria, a decrease in gastric *Helicobacter* and an increase in total bacteria in the duodenum [44]. In humans, PPI administration also alters the gut microbiota composition but results in a different profile [45,46]. Jackson et al. reported a lower abundance of Firmicutes and higher abundance of Bacteroidales in PPI users [46].

Other changes in the composition of the gut microbiota community following exposure to xenobiotics were reviewed by Lu et al. [47].

In addition to the administration of xenobiotics for medical reasons, humans and pet dogs are exposed to environmental chemicals that affect the host and resident gut microbiota on a daily basis. In this context, the study of Koestel et al. evidenced an effect of bisphenol A, an endocrine-disrupting chemical widely present in food-can linings, on the bacterial gut composition in pet dogs [48]. The authors identified disturbances in many bacterial genera and species, including *Bacteroides* spp., *Clostridium hiranonis*, *Bacteroides uniformis*, *Ruminococcus* spp., *Roseburia* spp., *Megamonas* spp., *Fusobacterium* spp., *Ruminococcus* spp., *Pectinatus* spp., *Catenibacterium* spp. and *Faecalibacterium prausnitzii*. According to the authors, the effects of bisphenol A could be extrapolated to humans, and therefore, dogs are considered to be bio-sentinels for human health concerns.

## 4. Pet Dog and Human Gut Microbiota in Disease Conditions

The gut microbiota is essential in the gastrointestinal homeostasis of animals and humans. Numerous studies have reported an association between the imbalances in the intestinal microbial ecosystem of pets and humans, also known as dysbiosis, with the presence of several inflammatory and endocrine diseases. The gut microbiota is also involved in extraintestinal diseases such as obesity, atopic dermatitis and diabetes mellitus [49].

### 4.1. Inflammatory Bowel Diseases

Inflammatory bowel diseases (IBDs) have emerged as a global health problem [49,50,51]. Similarly to humans, dogs also develop IBD as a result of genetic and environmental factors, aberrant immune responses, and the gut microbiota [14,52]. Unlike in humans, little is known about canine gut microbiota in IBD, and most observations originate from the analysis of fecal samples using 16S rDNA gene sequencing on limited numbers of dogs. The comparison of microbial populations from the studies available in dogs show noticeable variability among studies, and this may be related to numerous factors such as differences in the methodology (e.g., total headcounts, DNA extraction method and primer bias), age, breed, diet, geographical origin and housing environment of the studies together with the disease stage and medications.

**Figure 3:**
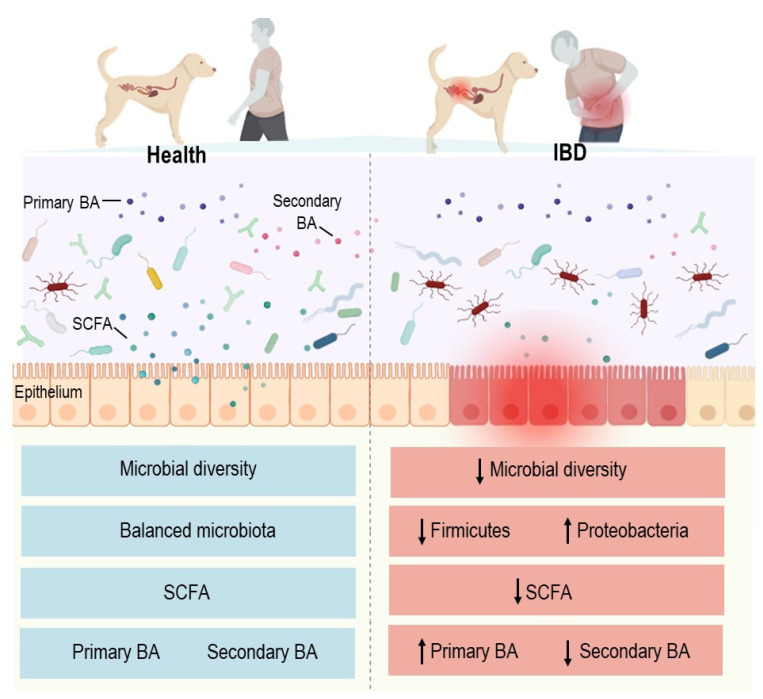
Humans and pet dogs show similar gut microbiota disturbances during IBD, characterized by a reduction in microbial diversity, a reduction in Firmicutes and an increase in Proteobacteria; a reduction in short-chain fatty acids (SCFAs); an increase in primary bile acids (BAs); and a reduction in secondary BAs.

**Figure 4:**
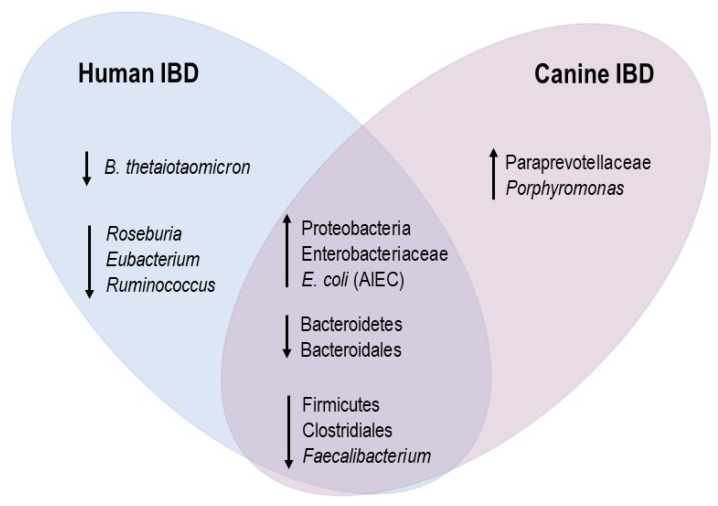
Bacterial features associated with human and canine IBD.

Alterations in the gut microbiota composition and function are also associated with canine IBD [53] (Figure 3). Similarly to human patients, dogs with IBD have a decreased microbial richness and diversity compared to healthy subjects [54]. Some deviations in the gut microbiota are common between humans and dogs with IBD, whereas others are host-specific [13]. Common modifications are characterized by both a superabundance of Enterobacteriaceae and Proteobacteria and a decline in Bacteroidetes and Firmicutes in the majority of subjects [55,56,57,58]. A decline in *Faecalibacterium prausnitzii* is reported in both humans and dogs with IBD in comparison to healthy individuals [13,59,60,61]. More specific changes include the overrepresentation of species of the Paraprevotellaceae family and *Porphyromonas* genus in dogs with IBD. Adherent-invasive *E. coli* (AIEC) is found in Crohn’s disease (CD) and ulcerative colitis (UC) patients [62,63,64] and in dogs with granulomatous colitis [65] (Figure 4). This breed-specific canine disorder has been described in Boxer and French Bulldogs and is sporadically reported in other breeds. The clinical and histopathological features are similar to those of CD and parallel to those of Whipple’s disease in humans [66,67]. Membranous colitis induced by *Clostridioides difficile* infection has been extensively studied in humans [67]. In dogs, the clinical involvement of *C. difficile* has not been established, given there is a prevalence of between 30 and 36% in the stools of asymptomatic dogs [68,69].

Dysbiosis induces metabolic alterations, including changes in SCFA and tryptophan metabolite production, which may affect gut homeostasis and immunological tolerance [70,71]. Increasing evidence demonstrates that the abundance of SCFA-producing bacteria dramatically decreases in fecal samples from human IBD patients, leading to reduced levels of SCFAs in the gut and the exacerbation of intestinal inflammation [68]. In dogs with chronic enteropathy, the fecal concentrations of acetate and propionate are also lower than those in healthy dogs [70]. As previously mentioned, the representation of the SFCA-producing bacterial phylum Bacteroidetes is decreased in human and canine IBD patients [70,71,72,73]. Other SFCA-producing bacteria, including some strains of *Faecalibacterium* spp., *Roseburia*, *Eubacterium* and *Ruminococcus*, are also reduced in human and canine IBD patients [70,71,72,73,74,75]. Butyrate is known to inhibit neutrophil recruitment, restore intestinal barrier function and alleviate the clinical and pathological features of *Clostridioides difficile*-induced colitis in mice [76,77].

The biotransformation of bile acids (BAs) by colonic microbiota is also involved in the pathogenesis of IBD in humans and pet dogs. The secondary bile acids (deoxycholic acid (DCA), lithocholic acid (LCA) and ursodeoxycholic acid (UDCA)) exhibit intestinal anti-inflammatory properties as demonstrated in vitro and in vivo in rodent models [78,79]. However, the formation of secondary BAs involves a deconjugation step mediated by the microbial bile salt hydrolase (BSH), followed by 7-alpha-dehydroxylation and epimerization supported by the colonic microbiota [80]. BSH has been identified in many bacterial genera, including *Lactobacillus*, *Bifidobacterium*, *Clostridium*, *Bacteroides*, *Faecalibacterium* and *Enterococcus* [78,81]. The conversion to secondary BAs is attributed to a smaller number of bacteria with bile-acid-inducible enzymes, including *Clostridium cluster XIVa* and *Eubacterium* among the genera of the phylum Firmicutes, whose populations are reduced during the course of human and canine IBD [81,82]. A preliminary study using untargeted metabolomics on fecal specimens from dogs with IBD confirmed increased primary BAs and decreased in secondary BAs in comparison to healthy dogs [61]. This pattern for secondary BAs in dogs with IBD was confirmed by two other studies [83,84], and the findings were similar to those of numerous reports on CD and UC [69,85].

Altogether, the data published for humans and dogs show a reduction in the diversity and richness of the intestinal microbiota with the progression of IBD. Functionally, the conversion of primary bile acids to secondary bile acids and SCFA synthesis by the gut microbiota appear to be similarly impaired in both species. Further studies with a standardized methodology and sufficient sample sizes are needed to compare the characteristics of the gut microbiota in humans and dogs with IBD.

### 4.2. Diabetes Mellitus

In recent years, changes in gut microbiota composition were suggested to be a potential contributor to type 2 diabetes mellitus (DM) [86]. While much of this understanding comes from studies in mice, which have highlighted the influence of the gut microbiota on glucose homeostasis, alterations in gut microbiota composition have also been noted in pet dogs, with DM. Jergens et al. observed intestinal dysbiosis and altered fecal bile acid (BA) levels in dogs with insulin-dependent DM, found to be similar to humans with T2DM [87]. Bacteria from the Enterobacteriaceae family were more abundant in diabetic dogs, whereas those from the Erysipelotrichia class and from Mogibacteriaceae and Anaeroplasmataceae families were overrepresented in healthy controls. At the species level, the proportion of an unclassified bacterial species from the Enterobacteriaceae family was most significantly correlated with DM, whereas the abundances of *Bacteroides plebeius* and *Lactobacillus reuteri* was associated with healthy individuals. Dogs suffering from DM had higher levels of total primary fecal unconjugated BAs in comparison to healthy dogs. The level of cholic acid was increased in the feces of diabetic dogs relative to controls. The link between BAs and host–microbiota interactions appears to be complex and bidirectional. Several pathways are involved in the microbial metabolism of BAs, including BA deconjugation by bacterial species possessing bile salt hydrolase activity and the generation of iso-BA by bacteria-producing hydroxysteroid dehydrogenases [88].

In humans, the relative abundance of Firmicutes, for instance, and Clostridia was significantly reduced in the diabetic group, compared to in healthy controls, in a study by Larsen et al. [89]. The ratios of Bacteroidetes/Firmicutes and the *Bacteroides–Prevotella* group to the *Clostridium coccoides–Eubacterium rectal* group were positively correlated with plasma glucose concentrations [89]. Other differences included decreased abundances of *Roseburia* species and *F. prausnitzii*, which are known to be producers of SCFAs [86,89,90,91]. Butyrate provides energy to colonic epithelial cells and has the potential to increase insulin sensitivity and energy expenditure [92].

### 4.3. Obesity

According to the World Health Organization (WHO), approximately 39% of human adults are considered obese and overweight [93,94]. The etiology is related to various factors, and the gut microbiota continues to draw attention as an element that affects disease status.

In pets, overweight and obesity are also frequent conditions that decrease life expectancy and trigger several comorbidities such as insulin resistance, systemic arterial hypertension and osteoarthritis [95]. Several authors have reported decreased bacterial diversity in the fecal microbiota of overweight (OW) and obese (OB) dogs when compared to normal weight (NW) dogs [96,97]. Handl et al. identified a greater abundance of the phylum Actinobacteria and the genus *Roseburia* in OB dogs [98]. When comparing 17 NW, 27 OW and 22 OB dogs, Forster et al. showed that the Erysipelotrichi class was more abundant in OW compared to OB dogs, and this was essentially led by differences in the *Eubacterium* genus [99]. The Actinobacteria class was determined to be present at higher levels in OB dogs relative to NW dogs. At the order level, Bifidobacteriales were significantly less abundant and Aeromonadales showed a tendency to be more abundant in OW relative to OB dogs. Comparatively to OB dogs, NW individuals exhibited higher levels of Erysipelotrichaceae, Erysipelotrichales and Erysipelotrichi and also had a lower abundance of the order Bifidobacteriales. When studied as operational taxonomic units (OTUs), the genus *Blautia* was more represented in NW and OW dogs than in OB dogs, as was the Lachnospiraceae family and the *Eubacterium biforme* species. The Ruminococcus family was more relatively abundant in NW than OB dogs. OTUs within the *Prevotella copri* species and the *Clostridium* genus were more abundant in OW than OB dogs. The order Clostridiales was also shown to increase in research dogs subjected to long-term ad libitum feeding when compared with NW control dogs [98]. Other authors reported a higher abundance of *Fusobacteria,* and more specifically, of the species *Fusobacteria perfoetens* in OW dogs in comparison to LN dogs [97]. Another study identified a predominance of the phylum Proteobacteria in OB dogs [96]. From a functional perspective, they speculated that an enrichment of Gram-negative bacteria may be implicated in chronic low-grade inflammation in OB dogs via increased levels of intestinal LPSs [100]. Gram-positive bacteria could modulate inflammation. In fact, Kainulainen et al. reported a beneficial role for the canine indigenous strain *Lactobacillus acidophilus* LAB20 and its ability to attenuate LPS-induced IL-8 production in HT-29 cells [101].

The impacts of dietary intervention and exercise on the canine gut microbiota have been investigated in several clinical trials [102,103]. Kieler et al. did not show any effects of exercise on the gut microbiota composition during a weight-loss program based on a commercial low-fat, high-protein and high-fiber, dry diet [103]. A negative correlation between the abundance of *Megamonas* and weight-loss rate was identified. The relative abundance of Ruminococcaceae was significantly lower at the end of the trial and the mid-term fecal concentrations of acetic and propionic acid were lower in dogs with rapid weight loss compared to dogs with slow weight loss. These data suggest that obese dogs exhibiting fecal bacteria that are able to produce acetic and propionic acids may be less amenable to weight loss due to an increased ability to extract energy from the diet through the production of SCFAs.

In humans, it was demonstrated that obese people present a lower diversity and richness in gut microbiota composition [104,105]. Indeed, several studies highlight an increase in the Firmicutes/Bacteroidetes ratio in obese people compared to healthy individuals [97,99,100]. Recently, a study by Palmas et al. showed an altered abundance of several taxa belonging to Bacteroidetes (*Bacteroides*, *Rikenella* and *Parabacteroides*), Firmicutes (*Eubacterium*, *Ruminococcus* and *Streptococcus*) and Proteobacteria (*Escherichia*, *Enterobacter* and *Klebsiella*) [106]. To better characterize the extent of its contribution to the disease, some mechanisms were proposed. Metabolites, especially SFCAs, produced by gut microbiota can regulate host energy metabolism, thereby increasing de novo lipogenesis in the liver and lipid accumulation in host adipocytes [107,108,109]. Studies of obese people have demonstrated a positive correlation between fecal SCFA concentrations and obesity [110,111]; however, others have reported a negative relationship between SCFA levels and the obese phenotype [112]. Moreover, Morrison and Preston postulated that SCFAs constitute signaling molecules mediating crosstalk between the host and its corresponding gut microbiota [108]. Hence, changes in SCFAs are representative of major carbon fluxes from the diet through the gut microbiota to the host, which serves as evident of their regulatory role in the overall metabolism [113]. Another proposed mechanism includes an increase in lipopolysaccharides (LPSs) produced by gut microbiota [93]. Indeed, LPSs can affect intestinal permeability, leading to an increase in their plasmatic concentration, which is correlated with the chronic low-grade inflammation characteristic of obese human patients. Additionally, it is well known that LPSs are able to bind to Toll-like receptor-4 (TLR-4), which upregulates the production of inflammatory cytokines and chemokines [114].

### 4.4. Kidney and Urinary Tract Diseases

The link between the gut microbiota and chronic kidney disease (CKD) has been investigated in large human cohorts showing a significant reduction in microbial diversity compared to healthy controls. The bacterial communities are distinct with an enrichment of the genera Akkermansia, Klebsiella and Enterobacteriaceae and a depletion of the genera Blautia and Roseburia in patients with non-hemodialyzed CKD. Some functions are predicted upward such as the metabolism of tryptophan and phenylalanine and others are predicted downward such as the metabolism of arginine and proline during CKD [115,116]. Patients with end-stage kidney disease on peritoneal dialysis were less likely to have Bifidobacterium catenulatum, Bifidobacterium longum, Bifidobacterium bifidum, Lactobacillus plantarum, Lactobacillus paracasei and Klebsiella pneumoniae than healthy controls [117]. These populational and functional changes are suspected to be due to the exposure of intestinal bacteria to urea crossing the gut barrier leading to a selection of bacterial families containing urease, uricase or indole- and p-cresyl enzymes [118]. Unfortunately, there is no comparative data available for dogs.

Few data on the link between gut microbiota and urolithiasis in humans and dogs are available. In dogs, *Oxalobacter formigenes*, a bacterium that degrades oxalates, was shown to exhibit significantly contrasting prevalence between dogs with oxalate calcium uroliths (25%), healthy dogs belonging to a breed predisposed to oxalate calcium uroliths (50%), and healthy non-predisposed dogs (75%) [119]. The significant underabundance of *O. formigenes* in the human gut has also been reported in patients with calcium oxalate urolithiasis since the early 2000s [120].

Very recently published data for humans make it possible to establish a correlation between a decrease in the abundance of SCFA-producing bacteria and the formation of calcium oxalate nephrolithiasis [121]. Liu et al. showed that SCFAs have the ability to reduce the formation of stones via regulating the expression of the intestinal transporter SLC26A6 involved in oxalate excretion [121]. This communication route between gut microbiota and the host has not been studied in the context of lithiasic disease in dogs.

### 4.5. Neuropsychiatric Diseases

Understanding the microbiota–gut–brain axis, with the goal of identifying innovative therapeutic approaches for mental disorders in humans and dogs is a current challenge [116]. Laboratory animals, and especially germ-free and gnotobiotic mice, have been invaluable tools for proof-of-principle studies demonstrating the impact of the gut microbiota in the healthy development and homeostasis of the nervous system and in psychiatric diseases, autism spectrum disorder, schizophrenia, Alzheimer’s disease, Parkinson’s disease and stroke [117]. Communication between the gut microbiota and the brain takes place via endocrine, immune, humoral and nervous channels, with particular attention given to the vagus nerve [118]. Data published by Cummings et al. show that 96.4% of new drugs developed for treating Alzheimer’s disease as a result of basic research using mouse models fail in human clinical trials [122]. There is an urgent need for novel models that will allow scientists to better translate the findings to humans.

Dogs exhibit behavioral problems of interest for exploring the links between gut microbiota and host behavior. They could serve as a model of spontaneous pathologies for investigating new therapeutic pathways. In particular, Kirchoff et al. studied a cohort of 21 conspecifically aggressive dogs and showed that the composition of the gut microbiota differed between aggressive and non-aggressive dogs [123]. More precisely, Proteobacteria and Fusobacteria displayed greater relative abundances in samples from non-aggressive animals, whereas Firmicutes were more abundant in samples from aggressive dogs. The Fusobacteriaceae family and more specifically, the *Fusobacteria* genus, were more abundant in specimens from non-aggressive dogs, whereas the Lactobacillaceae family and more specifically, the *Lactobacillus* genus, were more abundant in specimens from aggressive dogs. Mondo et al. compared the gut microbiota of dogs with behavioral disorders (aggressive and phobic conditions) with that of healthy controls [124]. The gut microbiota structure exhibited a robust dissociation of the aggressive population that seemed to be driven by a greater abundance of classically subdominant taxa, such as *Blautia*, *Catenibacterium*, *Collinsella*, *Ruminococcus Dorea*, *Megamonas* and *Slackia*. In contrast, the phobic population showed an enrichment of the *Lactobacillus* genus which comprises well-documented GABA producers. The major determinants of the segregation of the normal-behavior population were *Bacteroides*, *Faecalibacterium*, *Fusobacterium*, *Phascolarctobacterium* and *Prevotella*, evidencing the preponderance of bacterial genera typically contributing to the gut microbiota of healthy dogs. Mondo et al. applied a machine-learning technique (random forest) to their genus-level dataset and established *Catenibacterium* and *Megamonas* as bacterial determinant factors of aggressiveness [125]. Regarding idiopathic epilepsy, a pilot study using 16S rRNA gene amplicon sequencing failed to identify differences in overall fecal bacterial patterns and did not show quantitative variations in *Lactobacillus* species between untreated epileptic dogs and paired healthy dogs from the same household [125].

## 5. Conclusions

As shown in this review, the physiologies of the domestic dog and human are more similar than those of the human and mouse. Thus, the dog has the potential to be a useful animal model. The pet dog may be utilized in the field of translational sciences to identify novel therapies and maximize clinical benefits for both species. To the best of our knowledge, there are few (or no) studies in the field of gut microbiota that have used dogs as a larger animal model for the assessment of treatments to improve human wellbeing or health.

The vast majority of canine studies to date have focused on the phylogenetic structure of the microbiota and, in most cases, the bacterial fraction of the canine microbiota. Although this will increase the complexity of microbiota analyses, the characterization of the whole microbe community residing in the canine gut—i.e., the bacteria and archaea, fungi and yeasts, and protozoans and viruses—along with their interplay and their dynamics over time is likely to be key for understanding the ecology and physiology of the microbiota. The microbiota’s phylogenetic structure is undoubtedly an important parameter, but it is becoming clearer that functional information has to be associated with the microbiome structure in order to decipher the basis of the symbiotic crosstalk between the host and its microbiota to identify active compounds or species in addition to obtaining hints regarding the mode of action that determines the success of interventions.

Another challenge is to define what a healthy (or unhealthy) microbiota is and when appropriate, to evaluate whether microbiota or microbiota-produced compound(s) could help to better characterize a population of patients in order to develop efficient diagnostic tools using a combination of microbiota-based marker(s). It is likely that the meta-analyses of datasets, provided that the procedures are described and correspond to standards and best practices, would allow overcoming the limited number of dog samples included in most of the published studies. This raises the question of how the scientific community can develop and regularly update the required/useful best practices, standards and reference materials and define the type of information that has to be disclosed with the datasets. On these latter aspects, several international efforts are in progress, as are more specific initiatives. This question of larger numbers of samples and diversity of dog environments also highlights the potential of large international collaborative projects that would allow overcoming some of the putative biases associated with microbiome/population studies and determining whether local or worldwide scales will have to be considered for further developments in research and innovation.

## Figures and Tables

**Table 1:** Comparative physiology of humans, dogs, mice and pigs.

	Human	Pet Dog	Mouse	Pig
Lifestyle	Sedentary, active, athletic	Frequently similar to its owner	Standard laboratory accommodation	Standard laboratory accommodation
Environmental exposure	Domestic environment	Domestic environment	Laboratory environment	Laboratory or farm environment
Diet	Omnivorous	Omnivorous	Vegetarian omnivores	Omnivorous
Diseases	Spontaneous diseases (IBD, obesity, diabetes mellitus, etc.)	Spontaneous diseases similar to those in humans (IBD, obesity, diabetes mellitus, etc.)	Induced models of IBD, obesity, diabetes mellitus, etc.	Induced models of IBD, obesity, diabetes mellitus, etc.
